# Blending Better Beverage Options: A Nutrition Education and Experiential Workshop for Youths

**DOI:** 10.1155/2015/351734

**Published:** 2015-03-22

**Authors:** Kathy K. Isoldi, Veronika Dolar

**Affiliations:** ^1^Department of Nutrition, Long Island University, 720 Northern Boulevard, Brookville, NY 11548, USA; ^2^Department of Economics, Long Island University, 720 Northern Boulevard, Brookville, NY 11548, USA

## Abstract

*Objective*. To reduce intake of sugar-sweetened beverages (SSBs) in youths as a means to reduce obesity risk. *Methods*. Youths 5–14 years old attending a summer program were given a two-hour workshop addressing the sugar content in SSBs, the health risks from drinking SSBs, and hands-on preparation as well as tastings of low-sugar beverage alternatives. Data on usual intake of SSBs was obtained at baseline, and pre- and postprogram surveys were conducted to gauge change in knowledge and/or attitudes regarding SSBs. *Results*. There were 128 participants (63% male) in the program. SSBs were commonly consumed with over 80% reporting regular consumption (mean daily intake 17.9 ounces). Significant increase in knowledge regarding the sugar content of commonly consumed SSBs was achieved; however change in attitudes was not significant. The large majority of youths reported enjoying the workshop and intention to reduce intake of SSBs following program participation. *Conclusion*. SSBs are commonly consumed by youths. Knowledge regarding the sugar content of SSBs is easier to impart to youths than influencing attitudes held about these beverages. Long-term interventions that reach out to parents and address the widespread availability of SSBs are needed to influence resistant attitudes and beverage choosing behaviors in youths.

## 1. Introduction

An increase in the consumption of sugar-sweetened beverages (SSBs) and the prevalence of childhood obesity have occurred in tandem. In the United States (US) between 1977 and 2002 the increase in calories consumed from soft drinks and other sweetened beverages increased 230% and 170%, respectively [1]. Concurrently, the prevalence of childhood obesity increased threefold in the US, with those in minority and low-income groups experiencing higher prevalence rates [2, 3]. During this period, many other factors that increase obesity risk changed as well, such as an increase in sedentary activities, purchase of fast food, and sleep debt [4, 5]. However, SSBs are of special concern since the calories contained in this liquid form, for some reason, are not registered by the body, and therefore no dietary compensations are made following intake [6]. Instead paradoxically, researchers have found that when youths drink more SSBs it results in an increase in solid food consumption as well, with choices like pizza, burgers, and savory snacks often chosen [7]. Mathias et al. [7] found through analysis of data collected from the National Health and Nutrition Examination Surveys (NHANES) conducted between 2003 and 2010 that for every 100 kcalories of SSB consumed by 6–11-year-olds there was an increase in solid food consumed providing an additional 36 ± 14 kcalories. Youths 12–18 years of age revealed an intake of an additional 86 ± 10 kcalories in solid food form for every 100 kcalories of SSB product consumed. Recently, several reviews have illuminated the strong connection between obesity risk and SSB intake [8–10]; however weak potency of effect on interventions has called into question the absolute strength of this association [11].

In addition to an increased risk of obesity from consuming liquid calories, the high sugar content in SSBs has been associated with an increased risk of insulin resistance, dyslipidemia, type 2 diabetes mellitus, and cardiovascular disease [1, 6, 12]. Added sugars consumed from both liquid and solid sources are associated with body weight gain in youths at risk of developing obesity [6]; however Wang et al. [13] found in their group of 564 youths who were followed for two years that consuming sugar from liquid, but not solid, sources predicted a higher risk of developing impaired glucose homeostasis and glucose resistance. Reports spanning the past decade highlight increasing consumption of SSBs among children, adolescents, and teens [1, 4, 14, 15]. Recent estimates of the mean caloric contribution from SSBs range from 117 kcal/day to as much as 356 kcal/day, with calorie contribution variations based upon age category, sex, and ethnicity [14–17]. Those in minority and low-income groups have been identified as drinking greater amounts of SSBs [18]. It has also been found that approximately 5% of children and 16% of adolescents surveyed are heavy consumers of SSBs, with intakes at or exceeding 500 kcal/day [8]. SSBs are available to youths on the school campus as well as on the home front [17, 18]. However, researchers point to data supporting that the majority of SSBs are consumed at home [15, 17].

Public health experts have made a call for action in the form of educational interventions to address the excessive SSB intake in youths and subsequent adverse health issues [1, 9, 10, 14, 16, 17, 19]. The aim of the current study was to gauge impact on knowledge and attitudes regarding SSBs following a hands-on workshop for youths delivered during summer program hours. This experiential workshop addressed the sugar content of commonly consumed SSBs and included preparation and tasting of lower sugar alternatives. The study was given exemption status from Long Island University.

## 2. Methods

### 2.1. Setting and Participants

A convenience sample of youths who were enrolled in the summer program at a local boys and girls club participated in the nutrition education and blending better beverage options workshop. This program was provided to all attendees of the summer program at one boys and girls club in Long Island in New York State. All 128 summer camp participants were included in the workshop. Participants ranged in age from 5 to 14 years old. The workshop was delivered to approximately 20 participants at a time who were divided into small groups of 6–8 youths of similar age and were seated at one work table together with two undergraduate nutrition student volunteers.

### 2.2. Instrument

The survey instrument was developed by the study investigators and was based upon current literature [8, 16, 18] and designed to explore knowledge and beliefs about SSBs. The survey was modified to be age-appropriate; one version was created for 5–9-year-olds and another was developed for those who were 10–14 years of age. The same questions were asked, but the language was simplified and smile and frown faces were used for improved comprehension on the survey for the younger children. All participants were offered assistance with completion of the program surveys, and the younger participants were given one-on-one assistance when needed from undergraduate nutrition student volunteers. The survey was completed before the workshop began and following the end of the two-hour program for comparison.

#### 2.2.1. Usual Intake of SSBs

Each participant was asked to report their usual intake within four commonly consumed beverage categories (soda, sports drinks, sugar-sweetened tea and juice, and energy drinks) before the start of the workshop. For each category the participant was asked to estimate his/her frequency of consumption per week and then to estimate quantity consumed per frequency. Sample cans and bottles and representative glassware were displayed at each table to assist the participants in estimating the quantity of SSBs consumed. Nutrition undergraduate student volunteers assisted the participants in completing the survey.

#### 2.2.2. Knowledge of Sugar Content of SSBs

The survey included four questions regarding knowledge of sugar content (in teaspoon counts) of commonly consumed beverage items (16-ounce bottle of one-half sweetened iced tea and one-half lemonade, 12-ounce can of cola beverage, 20-ounce bottle of sweetened fruit punch, and an 8-ounce can of an energy drink). The participants were asked to select the amount (in teaspoons) of sugar from a list of four choices for each SSB item. The choices for each item were 3–5 teaspoons, 7–9 teaspoons, 10–12 teaspoons, or 15 or more teaspoons. The survey created for 5–9-year-olds included assistance in understanding the question by adding qualifying words for each selection with options listed as follows: 3–5 teaspoons,* a small amount*; 7–9 teaspoons,* a medium amount*; 10–12 teaspoons,* a large amount;* and 15 or more teaspoons,* a lot*.

#### 2.2.3. Attitudes Held regarding SSBs

To record and gauge any change in attitudes held regarding SSB preferences, thoughts about health concerns associated with SSBs, and intention regarding avoidance of SSBs, participants were asked to respond to six statements at baseline and again following the intervention. Following each statement, such as “*I should drink less soda and sweetened beverages*,” participants were directed to choose from a list of responses: strongly agree, agree, disagree, or not sure. The survey instrument completed by 5–9-year-olds included the following choices with accompanying faces to help them better understand and choose their response: yes definitely (broad smile), yes (smile), no (frown), and not sure (neutral).

#### 2.2.4. Postprogram Feedback

On the postprogram survey participants were asked to respond to a question asking whether they had enjoyed participation in the program. Attendees were asked to respond to the following statement: “*I've enjoyed participating in the beverage workshop*.” In addition, participants were asked to share their thoughts regarding intention to reduce intake of SSBs in the future by responding to the following statement:* “I think I will drink less sugar-sweetened beverages like soda because of what I've learned today*.” Once again the 10–14-year-old participants were asked to select from the following responses: strongly agree, agree, disagree, and not sure; and 5–9-year-olds chose their answer from yes definitely (broad smile), yes (smile), no (frown), and not sure (neutral), using visual faces to help them better understand and choose their response.

### 2.3. Intervention

Each participant took part in a two-hour workshop held during summer program hours that was composed of two separate, yet related, components.

1. Educational session revealing the sugar content of commonly consumed beverages and demonstration of adding a similar content of table sugar to water. Discussion of the health detriments associated with excessive sugar intake.
2. A hands-on, experiential involvement in blending better beverage options, followed by recipe tastings. A discussion about how to make healthier decisions for beverages.

Undergraduate nutrition student volunteers assisted participants in completing the program surveys and served as facilitators for the workshop. Each volunteer attended a one-hour instructional training session conducted by the Principal Investigator prior to the start of the program.

#### 2.3.1. Sugar Content Quiz and Demonstration

After completion of the baseline survey, each table of 6–8 participants took part in a guessing game and discussion about the sugar content of four popular beverage items led by an undergraduate nutrition student. Participants were asked to guess how many teaspoons of sugar were in each of four commonly consumed beverage items. After guessing, the nutrition student revealed the correct answer and asked the participants to count out sugar packets representing the amount of sugar contained in the item. A plate containing all the sugar packages was placed in front of the beverage item to offer a lasting visual image. This process was repeated for each of the four SSBs. When the process was completed for all beverages, the children were asked to view the four items on the table and to consider how much sugar would be consumed if all four SSBs were consumed in one day. The group added the total packages of sugar to achieve a grand total. Then the nutrition undergraduate students at each table led a demonstration showing how much sugar is added to liquid beverages by adding 15 teaspoons of sugar to a 20-ounce glass of water. This item was stirred and passed around for the participants to view the thick, cloudy substance that was created by simulating the amount of sugar often added to SSBs. An interactive discussion regarding the sugar content of SSBs and the health consequences of consuming too much sugar was held. Each nutrition undergraduate student was instructed to pose the following questions to the participants.

1. Is anybody surprised about the amount of sugar in these beverages?
2. Would anyone take a glass of water and add the same amount of sugar to it and then drink it?
3. Do you think drinking so much sugar in these types of beverages is harmful to your health?
  - Nutrition students were instructed to highlight the association of high sugar intake with weight gain, diabetes, and dental caries.
  - A review of the concerns associated with the ingredients in energy drinks and why children should not drink these products was conducted.
4. Would you like to make beverages to drink that are lower in sugar?

#### 2.3.2. Blending Better Beverage Options: Tasting and Discussion

Participants were led in a hands-on preparation of four recipes: (1) fresh peach and orange infused water, (2) pineapple, mango, peach, and lime slush, (3) cranberry, pineapple, and lime fizzy, and (4) fresh strawberry and banana smoothie. The participants had the opportunity to taste all items they had prepared. The nutrition undergraduate students were instructed to ask for participant feedback about the taste, acceptability, and ease of preparation of lower sugar beverage alternatives. The importance of preparing beverages using diluted versions of 100% fresh fruit juices was stressed.

### 2.4. Analysis

The study, including instruments, protocols, and consent procedures, received exempt approval by the Institutional Review Board at Long Island University. Written parent consent was not required because the student survey portion of this project was classified as exempt. Survey data results were tabulated and compiled into a database and analyzed using STATA (SE 13) to provide descriptive statistics and analysis. In addition to the standard Chi-square tests the analysis includes *t*-tests for comparing two population proportions. Proportions are among the few measures which can be used for summarizing categorical data and provide an additional dimension to the analysis. Unlike a Chi-square test that tests for the association between qualitative variables using the entire contingency table, the *t*-test can be applied to test, for example, if the proportion of participants correctly answering the question on the pretest is statistically different from the proportion of participants correctly answering the question on the posttest. The test statistic for comparing two population proportions is $t=\left(\left(\hat{p_{1}}-\hat{p_{2}}\right)-\left(p_{1}-p_{2}\right)\right)/\sqrt{\overset{-}{p}\left(1-\overset{-}{p}\right)\left(1/n_{1}+1/n_{2}\right)}$, where $\hat{p_{1}}-\hat{p_{2}}$ are sample proportions estimates, *p*_1_ − *p*_2_ are population proportions, and $\overset{-}{p}=\left(x_{1}+x_{2}\right)/\left(n_{1}+n_{2}\right)$ is the weighted average of the two sample proportion estimates. All *t*-tests results are one-tailed tests in order to study if one proportion of respondents is higher than the other, rather than simply being different from each other which would be captured by the two-tailed test. In other words, the tests are to assess if the proportion of participants correctly answering the question on the posttest is higher than the proportion of participants correctly answering the question on the pretest. Level of significance was set at *P* < 0.05.

## 3. Results

### 3.1. Participants

Specific participant sociodemographic data were not obtained due to the study's exemption status. However, study participants were attendees of the local boys and girls club afterschool program. The attendees of the program are predominately Latino and African American and come from single parent (51%) and low-income homes (76% come from families with incomes of less than $33,000/year and 74% receive free or reduced fee lunch). A total of 128 surveys were distributed to participants, 100% were returned, and there were no missing responses or surveys that were deemed incomplete. Of 128 participants, 81 (63.3%) were male and 47 (36.7%) were female, with an average age of 9.3 years. Data were analyzed using the entire sample of 128 participants as well as by two age subgroups: age of 5–9 years and age of 10–14 years. There were 70 participants in the 5–9-year-old age group (41 male and 29 female) with an average age of 7.6 years and 58 participants in the 10–14-year-old age group (40 male and 18 female) with the average age of 11.3 years.

### 3.2. SSB Intake

The average amount of SSBs consumed per week for the entire sample was 125.6 oz. (17.9 oz. per day), with 113.9 oz. (16.3 oz. per day) for the 5–9 year old age group and 139.6 oz. (19.9 oz. per day) for the 10–14-year-old age group. A two-sample mean comparison *t*-test found no statistically significant difference in total SSB consumption between the two age groups. In addition, the difference in drinking soda and sugar-sweetened teas and juices was not significantly different between the two age groups. However, the older group was found to drink significantly more sports drinks and energy drinks compared to the younger group (*P* < 0.05; Table 1). The drinking habits of males versus females in both the 5–9-year- and 10–14-year-old age groups were not statistically different. However, males in the 10–14-year-old age group reported to drink twice as much soda as females in this age group, 31.1 oz. and 15.6 oz. per week, respectively, and are significantly more likely to drink energy drinks, 45% and 11%, respectively (*P* < 0.05).

### 3.3. Knowledge of Sugar Content in SSBs

To evaluate the level of knowledge obtained by attending the beverage workshop pre- and postintervention survey data were analyzed using the standard Chi-square tests for the association between two qualitative variables. For the entire sample of 128 participants, the Chi-square test for all four knowledge questions rejects the null hypothesis even when the *P* value is set at *P* < 0.01. Since all the scores have improved, it can be concluded that the intervention was successful in providing information to the participants. The same conclusion is obtained for the 10–14-year-old age subgroup. However, for the 5–9-year-old age group, the Chi-square test failed to reject the null for improvement in knowledge on the question about the sugar content in an 8 oz. can of an energy drink.

**Figure 1:**
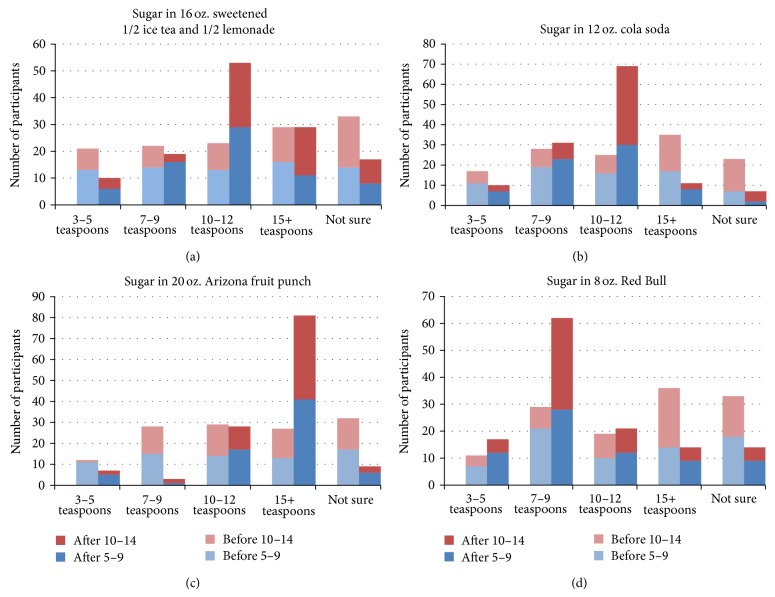
Baseline and postprogram SSB sugar content knowledge.

Results of the analyses of knowledge data using *t*-tests revealed that the proportion of participants who correctly answered the questions on the pretest for the entire sample of 128 participants is statistically different from the proportion of participants who correctly answered the questions on the posttest survey for questions 1 and 2 (sugar content in a 16 oz. serving of sweetened one-half iced tea and one-half lemonade; correct answer 10–12 teaspoons and in a 12 oz. can of cola soda; correct answer 10–12 teaspoons, resp.; Figure 1, panels (a) and (b)). However, for questions 3 and 4 (sugar content in a 20 oz. serving of sweetened fruit punch; correct answer 15+ teaspoons and an 8 oz. can of an energy drink; correct answer 7–9 teaspoons, resp., Figure 1, panels (c) and (d)), improvement in knowledge was increased, but not significantly. More precisely, for the age group of 10–14 years the scores on all four questions improved, while for the age group of 5–9 year olds only the scores for questions 1 and 3 (sugar content in a 16 oz. serving of sweetened one-half iced tea and one-half lemonade and the 20 oz. serving of sweetened fruit punch, resp., Figure 1, panels (a) and (c)) improved significantly.

### 3.4. Attitudes regarding SSB Intake

**Figure 2:**
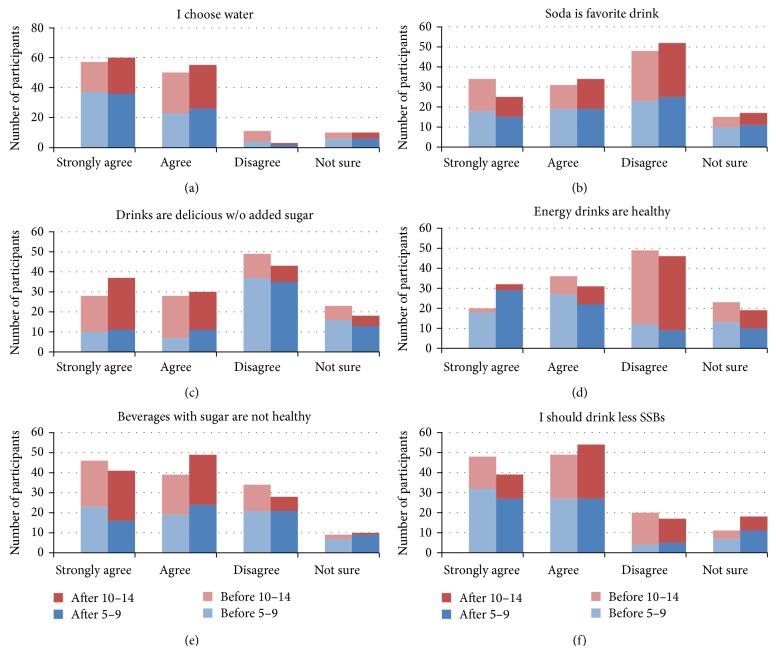
Baseline and postprogram responses to attitudinal statements.

A great majority of participants either strongly agreed or agreed with the statement that they usually choose a glass of water when they are thirsty, that beverages with sugar are not good for them, and that they should drink less soda and sweetened beverages (Figure 2, panels (a), (e), and (f), resp.), both before and after intervention. In addition, most participants disagreed with the statement that soda is their favorite drink, that delicious drinks can be made using fresh fruit and beverages without added sugar, and that energy drinks are healthy (Figure 2, panels (b), (c), and (d), resp.). The differences between the pre- and postintervention responses to comments addressing attitudes, however, are not statistically different when Chi-square tests were applied. In all attitudinal comments posed study participants responded favorably regarding attitudes held on the preprogram survey except when responding to the comment that delicious drinks can be made using fresh fruit without added sugar. The majority of participants disagreed with this comment on the preintervention survey, and although there was an increase in the number of those who strongly agreed or agreed with this comment following the intervention, there was no statistically significant change in response following program completion.

Responses to one comment went in an unexpected direction for the comment addressing whether an energy drink was a healthy beverage option. The majority of participants disagreed that an energy drink is a healthy beverage option on both the pre- and postintervention surveys; however, unexpectedly less rather than more participants disagreed with the statement after intervention compared with the preprogram surveys. This result was mostly driven by the change observed in the younger participants in the 5–9-year-old age group, where more of them either strongly agreed or agreed that energy drinks are healthy for them after the intervention. One possible explanation for this might be due to their unfamiliarity with this type of SSB.

## 4. Discussion

This interactive workshop conducted during summer program hours held with youths 5–14 years of age queried usual intake of SSBs and focused on transmitting knowledge about the amount of sugar contained in commonly consumed beverage items and the potential health detriments associated with overconsumption. The workshop also included a hands-on preparation and tasting of several lower sugar beverage alternatives. In agreement with current literature [8, 14, 15], the youths attending this workshop reported regular consumption of SSBs. We found that the large majority of the youths who participated in the workshop reported regularly drinking soda (87%), sports drinks (85%), and sweetened teas and juices (81%). Approximately one-quarter of the participants (24%) reported drinking energy drinks, with a significant difference in consumption found in those 5–9 years of age (16%) in comparison with those 10–14 years old (35%). This finding is not surprising given the age of our participants as energy drinks are more commonly consumed by teens and young adults. However, there are many health concerns associated with consuming energy drinks and young children can be influenced by the intense marketing efforts for these products [20, 21]. Therefore, reports of any intake of energy drinks in youths 5–14 years of age are of concern and require further investigation focused on this specific group of SSBs. Of significance is that males were four times more likely than females to consume energy drinks. Based on our results 10–14-year-old males, in comparison with younger children and females, were more likely to consume energy drinks and should be targeted in future interventions aimed at eliminating consumption of this problematic beverage in at-risk youth populations.

Estimated mean intake of soda, sports drinks, sugar-sweetened drinks, and energy drinks per week, rounded to the nearest ounce, was 26, 43, and 44 ounces and 1 ounce, respectively, for 5–9-year-olds and 26, 63, 41, and 4 ounces, respectively, for 10–14-year-olds. We found that the mean intake of fluid ounces of SSBs in our group of participants translated into approximately 17.9 ounces of beverage per day, or a little over two 8-ounce servings. This quantity of SSB translates into approximately 224 kcalories. The mean calorie contribution from SSBs found in this study is similar to what others have reported as usual calorie contribution from SSB in children and adolescents [14–17]. Noted in our data, and in agreement with current trends reported, sports drinks are being consumed with increasing frequency [8]. Researchers have reported that parents in Latino communities may exhibit a misunderstanding regarding sports drinks as some have been found to report that they believe that these drinks are healthy options for their children [18]. This misunderstanding in a community at increased risk of obesity and glucose intolerance is concerning and can promote future health risks. Therefore, outreach to parents, particularly in Latino communities to inform them about the health risks regarding the sugar content of sports drinks, appears advisable [18].

Chi-square tests on the entire sample of participants revealed a significant improvement in knowledge of sugar content for all four commonly consumed beverage items (iced tea/lemonade mix, cola beverage, sweetened fruit punch, and energy drink) between baseline and end of program. Since all the scores improved we conclude that the intervention was successful in providing information to participants.

Results of *t*-test analysis revealed improvement in knowledge after intervention for all four questions for 10–14-year-olds, but significance for 5–9-year-olds was only established for questions about the sugar content of iced tea/lemonade mix and sweetened fruit punch. These results are expected, since the participants in the age group of 10–14 years are more capable of understanding and retaining learned information. In addition, the older participants are also more likely to either consume and/or be familiar with cola beverages and energy drinks. In other words, the younger participants did improve their knowledge regarding the sugar content in all SSBs; however, for the sugar content in a 12 oz. can of cola and an 8 oz. can of an energy drink; correct answer 7–9 teaspoons, this change was not statistically different from the answers they provided in the pretest. It is not surprising that the younger aged participants did not remember the sugar content in energy drinks reviewed during the workshop as this is not a product that is widely consumed in this age category. It is possible that they did not register the information to memory due to lack of interest and familiarity with the product. Similarly, the younger participants would be more likely to drink sweetened fruit juice than cola beverages and this could explain the lack of significance found in change in knowledge for these beverage items.

Analyses of responses to six questions targeting attitudes about SSBs revealed no significant change in attitudes regarding beverage choice preference, thoughts about health concerns associated with SSBs, and wish to reduce SSBs following program participation. There are several factors that may explain the lack of change in attitude. One reason why there was not a considerable change found in participant attitudes might be that most participants already selected a favorable response prior to the intervention. Baseline surveys revealed that 85.7% of 5–9-year-olds and 81% of 10–14-year-olds reported that they choose water to drink when they are thirsty, and 84.3% of 5–9-year-olds and 65.5% of 10–14-year-olds reported that they knew that they should drink less SSBs. There were 57.1% of 5–9-year-olds and 74.1% of 10–14-year-olds who agreed that they thought beverages with added sugar were not good for them. However, it is possible that this comment may have been misunderstood by some who interpreted “not good for them” as not good in taste rather than not good for health. There were volunteers who read and explained the concept of the comment to the participants, but there may still have been a misunderstanding of the intention. Therefore, response results to this comment need further investigation and when the comment is posed to participants in the future clarity can be enhanced by instead asking for response to* “Beverages with added sugar are not healthy for me.*” Although not statistically significant impact of the program was evident as the results show that, following the intervention, more participants either strongly agreed or agreed with the statement that delicious drinks can be made using fresh fruit and beverages without added sugar (44.5% in the preintervention survey versus 52.3% in the postintervention survey). This trend in change of thought was particularly noticeable for the 10–14-year-old age group.

A significant percentage of children and adolescents seem to know they should reduce their intake of SSBs; however this knowledge does not translate into action. The preference for sweets is innate as well as learned, and so this biological response triggered by environmental availability may help to explain the resistance to behavior change in reducing SSB intake [22]. The strong desire for something sweet to drink and desire for a SSB in spite of known health risks were clearly stated by participants in a qualitative study conducted with college students [23]. College students are in an age category older than our participant sample and so even though they are more mature and should be able to better understand the risks of choosing to drink too many SSBs, the desire to drink what they wanted regardless was evident in the narrative captured by researchers [23]. Resistance to change in attitude and strong cravings for desired beverages make it difficult to see dramatic behavioral changes in attitudes held following short-term interventions. Despite these obstacles, Ebbeling et al. [24] report success in their study where they provided weekly deliveries of noncaloric beverages for 25 weeks to the homes of 53 children, aged 13–18 years. Compared to the control group the intervention group reduced their intake of SSBs by 84% and experienced a statistically significant reduction in body mass index (BMI) for those participants in the upper tertile for weight at baseline. Similarly, James et al. [25] conducted a year-long, school-based educational program for 644 schoolchildren in England who were 7–11 years old, called* Ditch the Fizz*, and found a small reduction in BMI in the intervention group and a modest reduction in consumption of carbonated drinks. Additionally, Sichieri et al. [26] report a statistically significant reduction in consumption of carbonated beverages in their seven-month-long, school-based intervention (*n* = 1134) with 9–12-year-olds that focused on increasing water intake. The intervention group received classroom activities and water bottles and had promotional banners hung at the school. A statistically significant reduction in BMI was found only in those who were overweight at baseline and only in females. Evidently, small successes in reducing SSB intake in youth are achievable. However, long-term interventions that address the home, school, and afterschool environments may be needed to realize greater impact.

There were several limitations with this study. A convenience sample was used for the study with all the participants coming from one boys and girls club in one community in the US. The participants were predominately Latino and African American and therefore study results cannot be generalized to other groups. In addition, data collected were self-reported and may be skewed due to participant bias or poor recall. Finally and in hindsight, the comment addressing thoughts about whether sweetened drinks were “not good” for the participant was found to be ambiguously worded and may have been misinterpreted. Study strengths include the use of trained nutrition undergraduate students to assist participants with survey completion and the interactive design of the intervention. We engaged youths in the learning process offering an experiential workshop that allowed the participants to prepare and taste alternatives to SSBs and also provided strong visuals to enhance impact and learning. We did not just tell youth participants to avoid SSBs but instead had them prepare and taste no- and low-sugar alternatives. The results from the program evaluation reveal that participants enjoyed participating in this program, as 87.5% either strongly agreed or agreed with this statement. In addition, almost 80% of the participants either strongly agreed or agreed with the statement* I think I will drink less sugar-sweetened beverages like soda because of what I learned today;* 81.5% in the 5–9-year-old age group and 77.6% in the 10–14-year-old age group (Table 2).

## 5. Conclusion

Childhood obesity and subsequent health detriment remain a formidable public health concern. Weight gain from consuming sugar in liquid form, such as in SSBs, is particularly concerning as liquid calories are not registered by the body and therefore not compensated for with subsequent reduction in food intake. SSBs are ubiquitous and they have made their way into the daily diet of children as they are readily available at home, on the school campus, and at afterschool venues. In this two-hour, hands-on intervention study we found that consumption of SSBs was common in 5–14-year-olds in three major categories: soda, sports drinks, and sugar-sweetened teas and juices. Energy drinks were less commonly consumed; however 24% of the participants reported consumption. Energy drinks should not be consumed by youths and interventions that address avoiding consumption of energy drinks in this age group are needed.

Despite providing a relatively brief intervention we were able to show a significant increase in participants' retention of knowledge regarding the amount of sugar added to commonly consumed SSBs. Postprogram data revealed that the large majority of participants enjoyed the program and intended to reduce intake of SSBs following participation in the program. However, we were unable to significantly influence attitudes held regarding SSBs. Long-term interventions and programs that engage youths and reach out to parents as well as addressing the widespread availability of SSBs are needed in the future to influence resistant attitudes and beverage choosing behaviors in youths.

## Figures and Tables

**Table 1:** Typical consumption of SSBs in youths attending a summer program.

Question	Response	5–9-year-olds number (%)	10–14-year-olds number (%)	Entire group number (%)
I drink soda				
Such as cola, ginger ale, Sprite, and Mountain Dew	Yes	60 (85.7)	51 (87.9)	111 (86.7)
	No	10 (14.3)	7 (12.1)	17 (13.3)
Mean times per week		2.37	2.09	2.25
Mean ounces consumed		9.91	11.1	10.45
Total mean ounces consumed per weeks		26.26	26.28	26.27
I drink sports drinks				
Such as Gatorade and Powerade	Yes	60 (85.7)	49 (84.5)	109 (85.2)
	No	10 (14.3)	9 (15.5)	19 (14.8)
Mean times per week		2.41	2.78	2.58
Mean ounces consumed		15.06	18.28	16.52
Total mean ounces consumed per weeks		42.54	63.03	51.83
I drink sugar-sweetened beverages				
Such as sweetened tea, fruit punch, and Sunny-D	Yes	57 (81.4)	47 (81.0)	104 (81.3)
	No	13 (18.6)	11 (19.0)	24 (18.8)
Mean times per week		2.84	2.81	2.83
Mean ounces consumed		12.16	11.98	12.08
Total mean ounces consumed per weeks		43.59	40.64	42.25
I drink energy drinks				
Such as Red Bull and Rockstar	Yes	11 (15.7)	20 (34.5)	31 (24.2)
	No	59 (84.3)	38 (65.5)	97 (75.8)
Mean times per week		0.3	0.78	0.52
Mean ounces consumed		1.49	3.94	2.6
Total mean ounces consumed per weeks		3.14	9.7	6.11
All beverages mean ounces		**113.87**	**139.64**	**125.55**

**Table 2:** Postprogram response: program evaluation and intention to change behavior.

	5–9 years number (%)	10–14 years number (%)	Total number (%)
*I enjoyed participating in the beverage workshop *			
Strongly Agree	43 (61.4)	32 (55.2)	75 (58.6)
Agree	17 (24.3)	20 (34.5)	37 (28.9)
Disagree	5 (7.1)	4 (6.9)	9 (7.05)
Not sure	5 (7.1)	2 (3.4)	7 (5.5)

*I think I will drink less sugar-sweetened beverages like soda because of what I learned today *			
Strongly Agree	34 (48.6)	19 (32.8)	53 (41.4)
Agree	23 (32.9)	26 (44.8)	49 (38.3)
Disagree	5 (7.1)	8 (13.8)	13 (10.2)
Not sure	8 (11.4)	5 (8.6)	13 (10.2)
